# Automatic exposure compensation in intraoral digital radiography: effect on the gray values of dental tissues

**DOI:** 10.1186/s12880-021-00733-x

**Published:** 2022-01-05

**Authors:** Evelyn Rute Carneiro Maciel, Eduarda Helena Leandro Nascimento, Hugo Gaêta-Araujo, Maria Luiza dos Anjos Pontual, Andrea dos Anjos Pontual, Flávia Maria Moraes Ramos-Perez

**Affiliations:** 1 Department of Clinical and Preventive Dentistry, Federal University of Pernambuco (UFPE), Av. Prof. Artur de Sá, 329-481, Recife, Pernambuco 52171-011 Brazil; 2 Present Address: Division of Oral Radiology, Department of Dentistry, Odontomed Imagem - Medical and Dental Services, Av. Dois Rios, 632, Ibura, Recife, Pernambuco 51.230-000 Brazil; 3 Oral Radiology Area, School of Dentistry, Federal University of Alfenas (UNIFAL-MG), R. Gabriel Monteiro da Silva, 700, Alfenas, Minas Gerais 37130-001 Brazil

**Keywords:** Dental radiography, Dental materials, Digital radiography, Digital system, Periapical radiography

## Abstract

**Background:**

This study aimed to investigate the effect of automatic exposure compensation (AEC) of intraoral radiographic systems on the gray values of dental tissues in images acquired with or without high-density material in the exposed region using different exposure times and kilovoltages. The influence of the distance of the high-density material was also investigated.

**Methods:**

Radiographs from the molar region of two mandibles were obtained using the RVG 6100 and the Express systems, operating at 60 and 70 kV and 0.06, 0.10, and 0.16 s. Subsequently, a titanium implant was inserted in the premolar’s socket and other images were acquired. Using the ImageJ software, two regions of interest were determined on the enamel, coronary dentine, root dentine, and pulp of the first and second molars to obtain their gray values.

**Results:**

In the RVG 6100, the implant did not affect the gray values (*p* > 0.05); the increase in kV decreased it in all tissues (*p* < 0.05), and the exposure time affected only the root dentine and pulp. In the Express, only enamel and coronary dentine values changed (*p* < 0.05), decreasing with the implant presence and/or with the increase in exposure factors. The distance of the implant did not affect the results (*p* > 0.05).

**Conclusions:**

AEC’s performance varies between the radiographic systems. Its effect on the gray values depends not only on the presence or absence of high-density material but also on the kV and exposure time used.

## Background

Digital image acquisition systems are increasingly present in dentistry due to their advantages compared to conventional films, such as requiring less exposure time and presenting the possibility of image enhancement, which avoid repetition and consequently reduce the radiation dose to the patient [1–3].

In some systems, image enhancement is also performed automatically by the software, after image acquisition, but before it is displayed on the monitor, through an image pre-processing tool called Automatic Exposure Compensation (AEC) [4–6]. This tool increases image contrast, modifying pixel values non-linearly, producing high-contrast radiographs [7, 8]. Also, AEC can modify the signal-to-noise ratio, presenting a lower value (i.e. darker image) compared to systems without AEC [7, 9]. Previous studies have shown that AEC can improve the accuracy of diagnosis of proximal caries in under- and overexposed images [4]. It was also stated previously that the effect of AEC is influenced by the presence of high-density objects [6, 10].

Galvão et al. [6], in an in vitro study, investigated the influence of the presence of high-density material, as well as its size and exposure time, on the gray values of radiodensity liquids equivalent to dental tissues. The authors concluded that a high-density material present in the image can influence the AEC adjustment, regardless of exposure time and radiographic system. However, the gray values of dental tissues were tested employing radiodensity equivalent liquids separately (one dental tissue at a time on each radiograph), which does not reflect the clinical condition. In addition, apart from radiographic systems and exposure time, other parameters may be applied for image acquisition, such as tube current and kilovoltage (kV), which are mainly responsible for the final image density and contrast, respectively. By applying different kV (i.e. different beam energy), AEC could act differently in adjusting the shades of gray, as X-rays beam with different energy reaches the image receptor.

AEC is an autonomous tool, and thus the pre-processing of the image cannot be controlled by the operator. Also, little is known about the real clinical effect of AEC on the gray values of dental tissues when high-density objects are present in the radiographed area, and the effect of the exposure parameters. Therefore, it is essential to understand its functioning and how it can be influenced by factors present in the radiographed area in clinical situations (i.e., presence of high-density materials) and technical parameters (i.e. kV and exposure time).

The aim of the present study was to evaluate, in an *ex vivo* model, the effect of AEC on dental tissues (enamel, crown dentine, root dentine, and pulp), using two different radiographic systems, under different acquisition parameters (exposure time and kV). In addition, the potential influence of the distance from the high-density material on the gray values was tested. The null hypothesis was that the effect of AEC does not influence the gray values of dental tissues, regardless of the variables considered in the study.

## Methods

This study was approved by the Research Ethics Committee of the Federal University of Pernambuco (protocol #3.594.302). All procedures performed were under the ethical standards of the Institutional and National Research Committee and with the 1964 Helsinki Declaration and its later amendments or comparable ethical standards. The informed consent requirement for this ex vivo study was waived by the Research Ethics Committee of the Federal University of Pernambuco, and permission to use the ex vivo sample was granted by this committee.

The study sample consisted of periapical radiographs obtained from two partially edentulous human mandibles belonging to the Oral Radiology Department used for teaching purposes, selected considering the following inclusion criteria: mandibles containing adjacent first and second molars on at least one side, and absence of the second premolar. Molars with dental restoration or endodontic treatment and mandibles with moderate to advanced bone loss in the molar region were excluded. Finally, two molar regions (one in each mandible) were available for the acquisition of the study images.

### Image acquisition

All images were acquired using the Heliodent Plus X-ray machine (Sirona Dental Systems, Bensheim, Germany) and two digital intraoral systems:

- A Complementary metal-oxide-semiconductor, direct radiography (DR) system, 12-bit depth—RVG 6100 (Kodak Dental Systems, New York, USA);
- A photostimulable phosphor (PSP) plate, computed radiography (CR) system, 14-bit depth—Express (Instrumentarium, Tuusula, Finland).

For both systems, the X-ray machine was set to operate at 7 mA (tube current not adjustable), two kilovoltages (60 and 70 kV), and three exposure times (0.06, 0.10, and 0.16 s). In each mandible, periapical radiographs of the right mandibular molars were acquired with and without the insertion of an 11 × 4 mm titanium dental implant (Titamax, Neodent, Brazil) in the alveolus of the second premolar, composing two experimental groups: control group (without high-density material in the radiographed region) and high-density group (with the dental implant in the radiographed region, placed within the edentulous socket of the right second premolar). Table 1 summarizes the study’s protocols and variables.

**Table 1:** Exposure protocols and study variables

Radiographic system	Group	kV	Time (s)
RVG 6100	Control	60 and 70	0.06–0.10–0.16
	High-density	60 and 70	0.06–0.10–0.16
Express	Control	60 and 70	0.06–0.10–0.16
	High-density	60 and 70	0.06–0.10–0.16

**Fig. 1:**
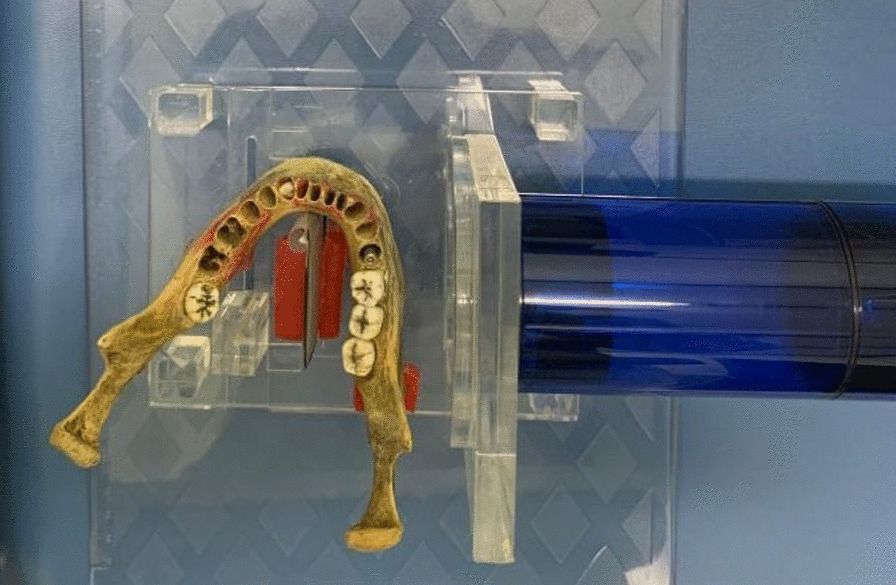
Acquisition of the radiographic image with the Express system (high-density group)

Images were acquired using the parallelism technique. An acrylic device was used to standardize the geometric relationships between the X-ray source/object/image receiver. Between the acquisitions of the control group and the high-density group, the mandible was not moved, so that the exact spatial relationship between it and the image receiver was kept the same throughout the study (Fig. 1).

Considering the objective nature of the analyses proposed in the present study, each acquisition was repeated 5 times [6]. The repetitions of acquisitions also aimed to verify the reproducibility of the method and record the possible variability of gray values between acquisitions. Thus, 240 radiographs were acquired (2 molar regions × 2 radiographic systems × 2 groups × 2 kV × 3 exposure times × 5 repetitions).

### Objective assessment of gray values of dental tissues

**Fig. 2:**
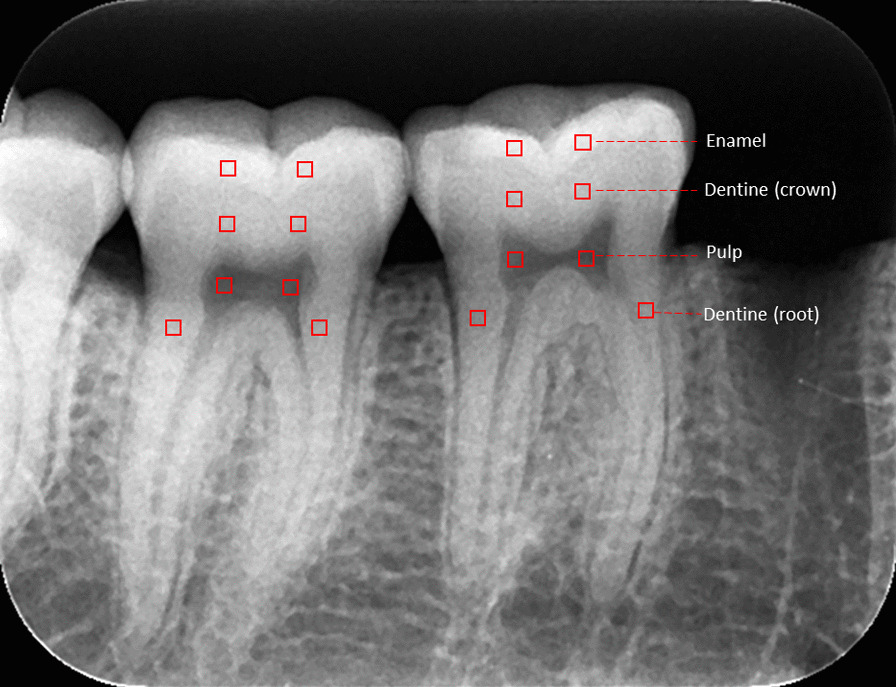
Representation of the square regions of interest located in the enamel, dentin (crown), dentin (root), and pulp of the first and second molars in an image obtained with the Express system

To have a standardization between the two systems used, all images were saved in TIFF format and exported with 8-bit depth to ImageJ software (U.S. National Institutes of Health, Bethesda, Maryland, USA). In the radiographs of each mandible, two regions of interest (ROIs) of size 0.65 mm × 0.65 mm were determined on the enamel, crown dentine, root dentine, and pulp of the evaluated teeth (Fig. 2). The ROIs were recorded in the ImageJ software using the ROI manager tool, which standardizes the ROI position on all images. In each ROI, the mean and standard deviation (SD) ​​of the gray values of each tissue were recorded.

### Statistical analysis

The mean and SD values of each tissue were tabulated in an Excel spreadsheet (Microsoft Corporation, Redmond, WA, USA) and analyzed using the SPSS software (v. 22.0, IMB Corp., Armonk, New York, USA). Two-way analysis of variance (ANOVA) was used to compare the gray values between the groups tested (control and high-density) with the varying protocol (kV and exposure time) within each tissue and radiographic system, followed by Tukey’s post-hoc test for multiple comparisons. An additional analysis was performed to evaluate the influence of the distance to the high-density material on the mean gray values. For that, the difference between the gray values (Δ gray values) in the high-density group and control group were calculated for the ROIs placed on enamel and dentine (both crown and root) of the first molar and the second molar, and compared by t-test. Those comparisons were performed only for those protocols that showed a statistically significant difference in the first part of the study. The significance level was set at 5% (α = 0.05).

## Results

The results related to the RVG 6100 system are shown in Table 2. For this system, the presence of the implant did not significantly affect the gray values of any of the dental tissues, regardless of the exposure time and the kV (*p* = 0.895). However, there were differences between the images acquired with 60 and 70 kV (*p* < 0.001): for all dental tissues, images obtained with higher kV showed lower gray values (i.e., darker image). By comparing the images obtained in the same kV with different exposure times, it was observed that the time affected only the gray values of the tissues of lower physical density (root dentine and pulp) (*p* < 0.001). In the control group, the increase from 0.06 to 0.16 s decreased the gray values of the root dentine at 70 kV and of the pulp at both kilovoltage (*p* = 0.026); in the high-density group, images acquired with 0.16 s showed lower gray values than the others, when 70 kV was used (*p* < 0.001).

**Table 2:** Mean and standard deviation (SD) of the gray values of dental tissues in the RVG 6100 radiographic system distributed according to the group, exposure time, and kilovoltage

Dental tissues	Group	t = 0.06 s		t = 0.10 s		t = 0.16 s	
		60 kV	70 kV	60 kV	70 kV	60 kV	70 kV
Enamel	Control	135.0 (12.7) Aa	118.1 (13.8) Ab	139.2 (11.9) Aa	118.2 (12.9) Ab	141.7 (13.2) Aa	115.4 (13.3) Ab
	High-density	134.8 (12.7) Aa	118.4 (13.1) Ab	139.6 (12.1) Aa	119.2 (13.2) Ab	142.2 (13.3) Aa	116.1 (13.7) Ab
Crown dentine	Control	112.2 (11.8) Aa	93.1 (11.4) Ab	114.3 (10.5) Aa	91.9 (10.3) Ab	115.4 (11.3) Aa	88.5 (10.5) Ab
	High-density	111.7 (11.5) Aa	93.1 (10.7) Ab	114.6 (10.5) Aa	93.0 (10.8) Ab	116.0 (11.4) Aa	89.1 (10.9) Ab
Root dentine	Control	97.3 (6.5) Aa	78.9 (6.3) Ab	98.5 (8.4) Aa	77.4 (7.8) Abc	99.2 (9.1) Aa	73.8 (7.9) Ac
	High-density	97.5 (6.1) Aa	79.3 Ab	99.6 (7.7) Aa	78.9 (7.1) Ab	100.5 (8.0) Aa	74.9 (6.8) Ab
Pulp	Control	49.7 (6.1) Aa	35.2 (5.1) Ac	47.2 (5.8) Aab	32.6 (4.3) Acd	46.2 (5.6) Ab	29.8 (4.1) Ad
	High-density	48.9(6.2) Aa	35.0 (5.0) Ab	47.7 (5.8) Aa	33.7 (4.7) Ab	47.0 (5.7) Aa	30.3 (4.1) Ac

Different uppercase letters indicate a statistical difference between the control and high-density groups, within each dental tissue (vertical comparisons)

Different lowercase letters indicate a statistical difference between kV at the same exposure time and between exposure times at the same kV, within each group (horizontal comparisons)

Table 3 details the results for the Express system, which shows that only the gray values of enamel and crown dentine underwent significant changes according to the study variables (*p* < 0.001). The presence of the implant caused a decrease in the gray values of both dental tissues at 70 kV, regardless of the exposure time (*p* < 0.001). The increase in kV decreased the values of crown dentine at 0.10 s and enamel at 0.10 and 0.16 s in the control and high-density groups, and of enamel at 0.06 s in the high-density group (*p* < 0.001). The increase in the exposure time from 0.06 to 0.16 s decreased the gray values of the enamel at both kV (*p* = 0.047) and of the crown dentine at 60 kV (*p* = 0.036).

**Table.3:** Mean and standard deviation (SD) of the gray values of dental tissues in the Express radiographic system distributed according to the group, exposure time, and kilovoltage

Dental tissue	Group	t = 0.06 s		t = 0.10 s		t = 0.16 s	
		60 kV	70 kV	60 kV	70 kV	60 kV	70 kV
Enamel	Control	227.5 (8.4) Aa	225.1 (10.3) Aab	226.0 (9.1) Aab	215.5 (10.1) Ac	220.5 (10.8) Ab	213.1 (10.8) Ac
	High-density	224.2 (10.4) Aa	217.2 (9.6) Bc	224.0 (10) Aab	208.5 (10.7) Bd	217.7 (11.5) Ab	205.4 (8.3) Bd
Crown dentine	Control	192.4 (13.4) Aa	190.5 (10.4) Aab	193.2 (11.6) Aa	181.0 (11.1) Ac	185.0 (8.8) Abc	185.2 (16) Abc
	High-density	187.7 (12.3) Aa	182.4 (9.7) Bab	189.5 (11) Aa	173.4 (8.4) Bc	180.2 (9) Abc	177.1 (11) Bbc
Root dentine	Control	172.4 (28.8) Aa	169.6 (22.4) Aa	171.9 (26.6) Aa	166.5 (21.4) Aa	167.7 (22.7) Aa	171.6 (26.4) Aa
	High-density	168.5 (25.3) Aa	162.7 (22.9) Aa	167.7 (25.2) Aa	158.4 (19.4) Aa	162.3 (21.5) Aa	164.1 (22.1) Aa
Pulp	Control	83.5 (24.3) Aa	95.6 (20.1) Aa	90.5 (24.5) Aa	98.1 (17.6) Aa	93.4 (20.8) Aa	92.4 21.5) Aa
	High-density	77.4 (22.4) Aa	89.4 (20.8) Aa	82.5 (23.2) Aa	93.6 (17.6) Aa	88.9 (19.8) Aa	88.3 (19.8) Aa

Different uppercase letters indicate a statistical difference between the control and high-density groups, within each dental tissue (vertical comparisons)

Different lowercase letters indicate a statistical difference between kV at the same exposure time and between exposure times at the same kV, within each group (horizontal comparisons)

**Fig. 3:**
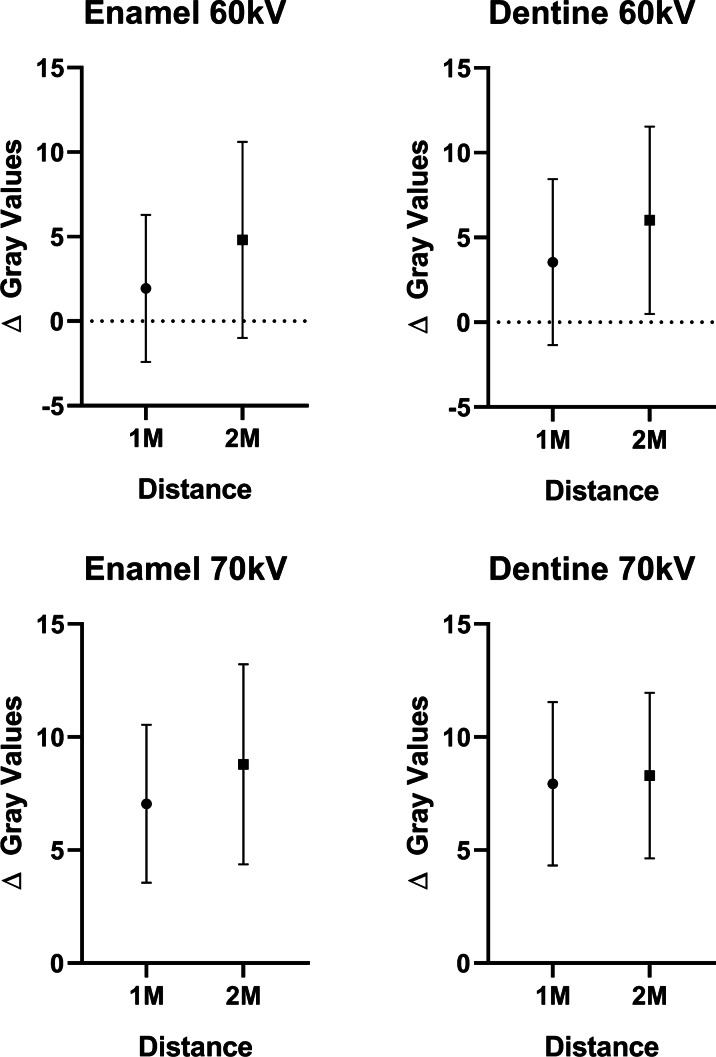
Graphs showing the mean Δ gray values and standard deviation between high-density and control groups for enamel and dentine of the first (1 M) and second molars (2 M) using 60 kV and 70 kV with an exposure time of 0.06 s for the Express system

The influence of the distance to the high-density material was tested only for those variables (Express system and kV) and dental tissues (enamel and crown dentine) that presented significant differences in the first evaluation. No difference was observed for the variation of the average gray values (Δ Gray Values) (control images—high-density images) between the first molar and second molar, in any of the kV and dental tissues tested (Fig. 3; *p* > 0.08).

## Discussion

It has been demonstrated here that the performance of the AEC depends not only on the presence or absence of high-density material in the radiographed area but also on the kV and exposure time used and the physical density of the radiographed anatomical structures. All these possible variables might influence on one system but not on the other, as the AEC can act differently for each digital system, as previously reported [6, 10].

In the RVG 6100 system, the presence of the implant did not significantly affect the gray values of the dental tissues in the exposure times and kV used. According to our knowledge, this was the first time that a system that present AEC did not show significant changes in any of the gray values of the structures present in the radiographed area when a high-density material was introduced into it, differently from what was found in other studies that also carried out similar evaluations [6, 10]. Such fact may be attributed to the fact that previous studies did not simulate a clinical scenario for this evaluation. The clinical disposition of the dental tissues and the attenuation of X-rays by bone and soft tissues covering (this latter represented here by an acrylic plate) may be factors that reduce the final effect of AEC activation by a high-density object.

In the Express system, only the gray values ​​of enamel and crown dentine change significantly, decreasing with the presence of the implant (at 70 kV) and/or with the increase in exposure factors. This result differs from the study by Galvão et al. [6] in which all materials equivalent to dental tissues (pulp, enamel, and dentine) were influenced by the presence of a high-density material (e.g., implant). In the present study, we classified dentin as “crown” and “root” because, although dentin has the same physical density throughout the entire tooth, its radiographic density could change as a result of overlapping enamel or alveolar bone depending on the region evaluated.

Previous studies that evaluated the effect of AEC in the presence of high-density material obtained varying results for each digital system. Galvão et al. [6] evaluated this effect in three different systems at 70 kV and demonstrated that, in two of them, Digora Toto (CMOS) and VistaScan (PSP), the presence of high-density material decreased the gray values ​​of all equivalent dental tissue materials, regardless of the exposure time; while in the third system, Digora Optime (PSP), there was a decrease only in the pulp equivalent tissue and there was an increase in dentine equivalent tissue for 0.1 and 0.16 s and an increase in enamel equivalent tissue in 0.16 s. Dashpuntsag et al. [10] also evaluated the Digora Optime system, at 60 kV, which demonstrated a similar operation to the Megadixel system (CCD) in the radiographs of a thin aluminum phantom: the addition of a high-density plate reduced the gray values and the contrast of the image. In the thick aluminum phantom, however, they presented opposite reactions: the gray values decreased in the Megadixel and increased in the Digora Optime system [10]. Therefore, it is noticed that the AEC is intensified by the presence of high-density material in the radiographed area can present different effects according to the digital system used. In addition, some of these systems, as in the case of Digora Optime, can have a bidirectional function, causing both increasing and decreasing gray values according to the exposed structure. It is worth emphasizing that these previous studies used phantoms with tissue-equivalent liquids representing each dental tissue individually [6] or aluminum scales [10] in their study designs, unlike ours that used real teeth and mandibles.

This was also the first study that evaluated the effect of the distance of the high-density material on the gray values of the adjacent dental tissues. This was achieved through a statistical evaluation of the difference of variations in gray values that occurred between the tissues of the first and second molars, with the introduction of the dental implant in the image. Thus, we observed that there was no significant influence of distance on any tissue. Differently, Galvão et al. [6] evaluated the size of the equivalent high-density material in the radiographed region, representing what would be a different amount of high-density material in the image, but also found a similar result: this factor did not affect the gray values ​​significantly. Even so, as these authors did not use dental tissues for radiographic acquisition, the variable “amount of high-density material” in the radiographed area still needs to be further studied.

Regarding kilovoltage, in the RVG 6100 system, the increase in kV from 60 to 70 decreased the gray values in all dental tissues and exposure times, thus being the parameter that most caused changes in the image. In the Express system, the increase in kV decreased the gray values ​​only for crown dentine and enamel (tissues of greater physical density). In theory, the increase in kV qualitatively modifies the X-ray photons, increasing their capability to pass through the tissues. In systems without the AEC, this also results, although indirectly, in the darkening of the radiograph (reduction of gray values), since more X-ray photons will reach the image receiver [5]. As this is expected when the AEC function is not present but also occurred in systems with AEC, it seems that the AEC does not interfere so strongly in the changes caused by kV in the image. Hence, it is shown the importance of taking kV into account when comparing studies and when translating these findings to clinical application. No previous study has demonstrated the influence of kV on AEC function, and this makes it difficult to compare our results.

The behavior of the radiographic systems studied was the opposite when we analyzed the results regarding the exposure time. In the RVG 6100 system, the increase in time decreased the gray values ​​of pulp and root dentine, while in the Express system, the increase in exposure time decreased the values ​​of enamel and crown dentine in some protocols. As is known, increased exposure usually causes the darkening of the entire radiograph. Thus, we could attribute to the AEC the fact that the tissues with higher and lower physical density were not affected in the RVG 6100 and Express, respectively. Dashpuntsag et al. [10] also evaluated the effect of the exposure time variation on radiographs from thin and thick aluminum scales. On the thin scale, the increase in time hardly caused changes in gray values in the Digora Optime system, while causing an important variation in Megadixel system. On the thick scale, increasing the time decreased the gray values ​​in Digora and increased in Megadixel. This indicates that the effect of the variation in exposure time in AEC systems depends on the thickness of the exposed structures and the radiographic system used.

In the present study, the images were evaluated on an 8-bit scale instead of the original scale of each system to obtain a standardization between them and to compare our findings with previous studies, which have also used that methodology. This scale presents a more homogeneous distribution of gray values over the 254 possibilities between black and white, while larger scales may group the values in a more heterogeneous way (which makes it more difficult to identify statistical differences in the variation of gray values). As for the use of the ImageJ software, it is a software that is widely used in radiological studies with methodologies similar to ours [6]. This software, in addition to precisely measuring the gray values in the chosen scale, also enabled us to standardize the size and location of the ROIs in the images, through its ROI Manager tool. Such an exact standardization would hardly be achieved in the software of each device.

The results obtained here are not sufficient to fully explain the action of the AEC in the gray values of the tissues, given that the AEC is an automatic tool whose performance is not disclosed by the manufacturers. According to Hayawaka et al. [8], the AEC function algorithm produces a high-contrast image irrespective of exposure, in a non-linear manner. Thus, the AEC algorithm could provide compensation for different degrees of exposure by stretching the range of pixel values to increase the image contrast, probably detecting the minimum and maximum pixel values of the image, redistributing and shifting these values to change the displayed image [8]. As AEC activity cannot be turned off by the operator, it is not possible to compare with the results of the same system without AEC. Our findings, however, clarify that the effect of AEC in a clinical situation differs from that in assessments of the radiographic density of liquids or aluminum scales [6, 10].

In addition, few studies have evaluated AEC from a clinical perspective, such as the effect of AEC on the accuracy of diagnosing proximal caries [4] and the repercussion of the presence of high-density material on this diagnostic task [11], which presented controversial results. Dashpuntsag et al. [10] also demonstrated that the alteration of contrast in the image caused by AEC can impair the accuracy of other diagnostic tasks, such as the detection of minimal bone regeneration in periodontal treatments, root cracks, and fractures. Likewise, the change of gray values by AEC can seriously affect the results of radiographic density surveys, especially those in which a clinical scenario is not simulated, such as in vitro studies of the density of types of cement and resins and other dental materials. Thus, the presence of AEC in radiographic systems can represent disadvantages in its use. Moreover, the X-ray unit used in the present study had a fixed tube current at 7 mA. The tube current, as the exposure time, is directly related to the number of photons produced and reaching the receptor. Therefore, one may expect that tube current and exposure time could impact the AEC intensity similarly. Future studies evaluating the effect of AEC on gray values of dental tissues in clinical conditions varying exposure parameters in other digital systems are encouraged. In addition, investigating AEC influence on other diagnostic tasks is also recommended.

## Conclusions

In conclusion, AEC’s performance varies between digital radiographic systems. Its effect on the gray values of dental tissues depends not only on the presence or absence of a high-density material in the radiographed area but also on the kV and exposure time used.

## Data Availability

The datasets generated and analyzed during the current study are available from the corresponding author on reasonable request.

## Abbreviations

AEC: Automatic exposure compensation
kV: Kilovoltage
ROI: Region of interest
SD: Standard deviation
